# Analysis of pelvic motion during gait with bivalve brace

**DOI:** 10.1186/1748-7161-9-S1-O50

**Published:** 2014-12-04

**Authors:** Lydie Journoud, Julie Deceuninck, Jean-Claude Bernard, Cyril Lecante

**Affiliations:** 1 Lecante SA, Lyon, France; 2 CMCR des Massues, Lyon, France

## Background

In the literature many articles explain that it is essential to have a femoral brace to restricted pelvic motion during gait. However, two principles to restrict the movement of the trunk in a brace: contention (hydraulic structure of the trunk) and the balance of power (three points system). But we are unable to know how much.

## Aim

The aim of this study is pelvis moves into the thoraco lumbar brace during gait, compared to a gait without the brace.

## Materials and methods

2 asymptomatic adults, with similar physical feature.

They wear a thoracolumbar bivalve brace, made with the same protocol (using CAD CAM) for reproducibility. We design the braces according to the principles of the brace immobilization:

- a good grip on the waist and overall tightening

- supports on the abdomen, thorax and lumbar.

We use the gait analysis system Vicon®. To use this system, we make holes in the brace at the location of the markers. Each person walks 6-8 trials in the gait analysis system with and without the brace.

## Results

The kinematic curves of the pelvic motion compared to the laboratory standard show that there is a significant decrease in the movement of the pelvis. With the brace, the range of motion is negligible.

## Conclusion

This preliminary experimentation allowed us to see that we could use the gait analysis system to evaluate the efficiency of brace. In a second step we have to consider a study on the patients who wear their braces on a longer time. So we will be able to objectify the efficiency of immobilization of the pelvis in a brace.

